# Effect of carotid corrected flow time combined with perioperative fluid therapy on preventing hypotension after general anesthesia induction in elderly patients: a prospective cohort study

**DOI:** 10.1097/JS9.0000000000000863

**Published:** 2023-11-16

**Authors:** Shishi Huang, Zhenqi Liao, Andi Chen, Jiali Wang, Xiaodong Xu, Liangcheng Zhang

**Affiliations:** a Department of Anesthesiology, Fujian Medical University Union Hospital, Fuzhou; b Department of Anesthesiology, Shengli Clinical Medical College of Fujian Medical University, Fujian Provincial Hospital, Fuzhou, People’s Republic of China

**Keywords:** carotid corrected flow time, Doppler ultrasound, elderly patients, general anesthesia, perioperative fluid therapy

## Abstract

**Background:**

Hypotension often occurs following the induction of general anesthesia in elderly patients undergoing surgery and can lead to severe complications. This study assessed the effect of carotid corrected flow time (FTc) combined with perioperative fluid therapy on preventing hypotension after general anesthesia induction in elderly patients.

**Materials and methods:**

The prospective cohort study was divided into two parts. The first part (Part I) consisted of 112 elderly patients. Carotid FTc was measured using Color Doppler Ultrasound 5 min before anesthesia induction. Hypotension was defined as a decrease of greater than 30% in systolic blood pressure (SBP) or a decrease of greater than 20% in mean arterial pressure (MAP) from baseline, or an absolute SBP below 90 mmHg and MAP below 60 mmHg within 3 min after induction of general anesthesia. The predictive value of carotid FTc was determined using receiver operating characteristic (ROC) curve. The second part (Part II) consisted of 65 elderly patients. Based on the results in Part I, elderly patients with carotid FTc below the optimal cut-off value received perioperative fluid therapy at a volume of 8 ml/kg of balanced crystalloids (lactated Ringer’s solution) in 30 min before induction. The effect of carotid FTc combined with perioperative fluid therapy was assessed by comparing observed incidence of hypotension after induction.

**Results:**

The area under the ROC for carotid FTc to predict hypotension after induction was 0.876 [95% confidence interval (CI) 0.800–0.952, *P*<0.001]. The optimal cut-off value was 334.95 ms (sensitivity of 87.20%; specificity of 82.20%). The logistic regression analysis revealed that carotid FTc is an independent predictor for post-induction hypotension in elderly patients. The incidence of post-induction hypotension was significantly lower (*P*<0.001) in patients with carotid FTc less than 334.95 ms who received perioperative fluid therapy (35.71%) compared to those who did not (92.31%).

**Conclusions:**

Carotid FTc combined with the perioperative fluid therapy could significantly reduce the incidence of hypotension after the induction of general anesthesia in elderly patients.

## Graphical abstract

**Figure:**
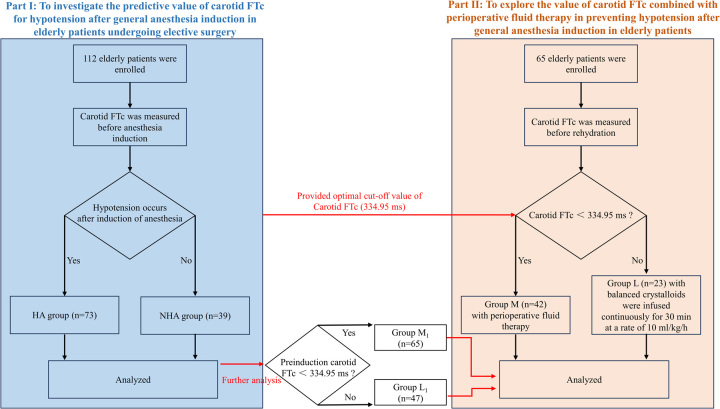

## Introduction

Highlights

- Hypotension following the induction of general anesthesia is common in elderly patients and can result in severe complications; however, effective predictors and interventions are scarce.
- Carotid corrected flow time (FTc) is a reliable predictor of post-induction hypotension in elderly patients; the probability of post-induction hypotension sharply increases in elderly patients with a carotid FTc less than 334.95 ms before induction.
- Among elderly patients with a carotid FTc less than 334.95 ms prior to induction of general anesthesia, administering perioperative fluid therapy at a volume of 8 ml/kg for 30 min significantly reduces the occurrence of post-induction hypotension.
- This article proposes for the first time that combining carotid FTc with perioperative fluid therapy is a potentially effective strategy for preventing hypotension after the induction of general anesthesia in elderly patients in clinical practice.

During the induction of general anesthesia, hypotension can easily occur as a result of blood pressure fluctuations caused by myocardial depression and vasodilation from general anesthetic drugs^1^. Additionally, the presence of preoperative disease states, dietary abstinence, and bowel preparations can create a relative deficiency in blood volume, further contributing to the development of hypotension^2,3^. Various studies^1,4–7^ have demonstrated that severe or prolonged hypotension following the induction of general anesthesia can lead to organ hypoperfusion and ischemia. As a result, this condition increases the risk of adverse postoperative outcomes, such as myocardial injury, ischemic stroke, or acute kidney injury. Furthermore, it has been observed that it may contribute to elevated 1-year mortality rates^8^. Even though hypotension following the induction of general anesthesia can be addressed with proper fluid replacement and vasoactive drugs. Once hypotension after general anesthesia happens, it often persists for a period and impacts the prognosis of patients. If the blood pressure drops excessively or persists for an extended duration, even if ultimately corrected, it can also significantly influence the patients’ prognosis and escalate the incidence of postoperative complications^7^. However, an effective method for accurately predicting post-induction hypotension and preventing it in advance in clinical practice is still lacking.

The population of elderly patients undergoing elective surgery has been progressively growing in recent years. The elderly patients often present with various cardiovascular conditions, including coronary artery disease, hypertension, and atherosclerosis, prior to surgery. Consequently, these patients are more susceptible to experiencing hypotension following the induction of general anesthesia, which poses an increased risk for anesthesia management. Additionally, this population manifests lower resilience to post-induction hypotension following general anesthesia, thereby resulting in an escalated risk of life-threatening complications, including severe acute myocardial infarction and acute kidney injury^7,9^. Therefore, the prevention of hypotension in elderly patients undergoing elective surgery following the induction of general anesthesia holds significant clinical value.

Previous studies^10–12^ pointed out that perioperative fluid therapy administered to surgical patients prior to the induction of general anesthesia has been found to effectively reduce the occurrence of post-induction hypotension while promoting more stable intraoperative circulation. However, elderly individuals are less tolerant of fluid overload, and implementing perioperative fluid therapy for patients without a fluid deficit can potentially lead to adverse effects due to fluid overload^13^.

Assessing fluid responsiveness is crucial during perioperative management, as improper fluid administration may lead to unfavorable outcomes^4^. Nevertheless, accurately evaluating a patient’s intravascular volume status during the perioperative period remains challenging. Traditional static indices, including central venous pressure and pulmonary capillary wedge pressure, have been critiqued for their lack of precision in predicting fluid responsiveness^14,15^. On the other hand, dynamic indices such as pulse pressure variation (PPV) that rely on heart–lung interaction have proved to be reliable guides for predicting fluid responsiveness in patients subjected to mechanical ventilation^16^. However, the ability of dynamic indices to assess fluid responsiveness during spontaneous breathing have largely been disappointing^17,18^. Furthermore, invasive vascular catheterization, necessary for the measurement of these indices, may present discomfort to conscious patients. Recently, ultrasonography has been widely advocated for estimating volume status, primarily due to its non-invasive characteristics, simplicity of data gathering, and the reproducibility of its measurements^19^. Among these ultrasound methods, the corrected flow time (FTc) measured in the carotid artery represents a novel strategy for predicting fluid responsiveness and has demonstrated encouraging outcomes^4,20,21^. Moreover, carotid FTc remains unaffected by respiration, making it a dependable static parameter for predicting fluid responsiveness in spontaneously breathing patients^22^. However, a limited number of studies^7,23^ have evaluated the clinical relevance of carotid FTc in predicting hypotension after the induction of general anesthesia in elderly patients.

Accordingly, the present study aimed to investigate the predictive value of carotid FTc for hypotension after general anesthesia induction in elderly patients undergoing elective surgery in the first part. Additionally, we conducted further research to explore the value of carotid FTc combined with perioperative fluid therapy in preventing hypotension resulting from blood pressure fluctuations after general anesthesia induction in elderly patients in the second part.

## Materials and methods

### Study population

This work has been reported in line with the STROCSS criteria^24^. This study received approval from the Medical Ethics Committee of our hospital and was registered in the ClinicalTrials Research Registry. Part I of this study, conducted between June and October 2022, involved the selection of elderly patients undergoing elective surgery under general anesthesia in our hospital. Immediately following this, Part II of this study was executed, involving the selection of elderly patients undergoing elective surgery under general anesthesia between October and December 2022. Both parts of this study adhered to identical inclusion and exclusion parameters. The inclusion criteria encompassed patients aged 65–85 years old, without discrimination of gender or surgical procedure, meeting ASA grades ranging from I to III. The exclusion criteria were as follows: (1) Patients with sudden and urgent preoperative changes in vital signs that are life-threatening. (2) Any history of neck surgery or trauma. (3) History of peripheral arterial disease or atherosclerosis. (4) Patients with BMI greater than 30 kg/m^2^ or less than 15 kg/m^2^. (5) History of cardiomyopathy or valvular heart disease. (6) Left ventricular ejection fraction less than 50%. (7) Patients in non-sinus rhythm. (8) Patients with implantable pacemakers or cardioverters. (9) Right ventricular dysfunction. (10) Chronic obstructive pulmonary disease. (11) Pulmonary hypertension. (12) Chronic kidney disease. (13) Patients with secondary hypertension. (14) Patient refusal. Similarly, patients who have blurred ultrasound measurements, sustained blood pressure exceeding 180/110 mmHg, temporary withdrawals, or encounter unanticipated difficult airways were excluded from the study. All participants provided informed consent, either personally or through their family members, by signing an informed consent form. All completed informed consent forms were collated and forwarded to the Clinical Trial Research Center at our hospital for documentation.

### Induction of general anesthesia procedure

A fast tract enhanced recover policy was implemented where all patients adhered to a standard regimen of fasting and fluid restriction that commenced 8 and 4 h prior to surgery, respectively^25^. Upon arrival in the operating room, vital parameters such as electrocardiogram (ECG), heart rate (HR), and pulse oxygen saturation (SpO_2_) were monitored. A radial artery puncture catheter was inserted for the continuous monitoring of the arterial blood pressure (IBP). Concurrently, a peripheral vein was accessed, and a balanced crystalloid solution (lactated Ringer’s solution) was routinely administered at a rate of 10 ml/kg/h. Subsequently, carotid FTc was evaluated using a carotid ultrasound. The induction of anesthesia was initiated 5 min later. For the induction of general anesthesia, intravenous administration included propofol at a dose of 1.5 mg/kg (with a push rate of ~40 mg/10 s), sufentanil at a dose of 0.4 μg/kg (with a push rate of about 4 μg/10 s), and cisatracurium at a dose of 0.15 mg/kg. Endotracheal intubation was performed 3 min after the administration of these medications.

### Study procedure

In Part I of this study, previous studies^4,10,26^ have shown that the area under the receiver operating characteristic curve (AUROC) of FTc for predicting hypotension after induction of general anesthesia in patients ranged from 0.82 to 0.91. Therefore, we assumed a minimum AUROC of 0.7, a significant difference (*a*)=0.05, and the power of a test (1−*β*)=0.95, while anticipating a 10% dropout rate. Using these parameters, the receiver operating characteristic (ROC) sample size computation within the PASS 15 software determined that Part I of this study would be necessary to enroll 112 patients. Ultimately, 112 elderly patients were enrolled and divided into hypotension group (HA) and non-hypotension group (NHA) according to the presence or absence of hypotension after induction, which was defined as a decrease of greater than 30% in systolic blood pressure (SBP) or a decrease of greater than 20% in mean arterial pressure (MAP) from baseline, or an absolute SBP below 90 mmHg and MAP below 60 mmHg within 3 min after induction of general anesthesia^10,27^. Baseline SBP, diastolic blood pressure (DBP), MAP, and HR were recorded before induction. Carotid FTc was measured using Color Doppler Ultrasound 5 min before anesthesia induction. SBP, DBP, MAP, and HR were recorded at 1, 2, and 3 min after induction, and the lowest value was taken. The threshold and predictive values of carotid FTc were determined using ROC curve and statistical analysis.

**Figure 1:**
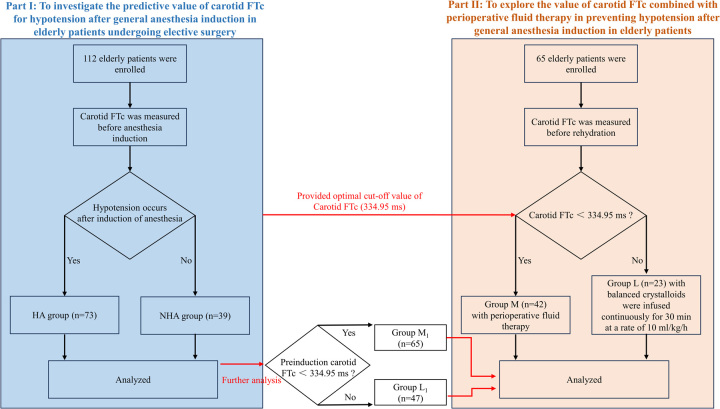
CONSORT flow diagram. FTc, corrected flow time; HA, hypotension group; NHA, non-hypotension group.

In Part II of this study, PASS 15 was used to estimate the sample size based on the incidence rates of hypotension after induction, which were 67.7% and 45% as observed in our Part I study and the pre-test of Part II study, respectively. Therefore, we assumed the significant difference (*a*)=0.05, and power of a test (1−*β*)=0.95, while anticipating a 10% dropout rate. The sample size was calculated to be 66. Ultimately, one patient was excluded due to persistently exhibiting an SBP higher than 180 mmHg, while the remaining 65 patients were incorporated into the analysis. Sixty-five elderly patients were divided into group M (carotid FTc <334.95 ms) and group L (carotid FTc ≥334.95 ms) based on pre-rehydration carotid FTc values. In group M, the patients received perioperative fluid therapy at a volume of 8 ml/kg of balanced crystalloids (lactated Ringer’s solution) in 30 min to compensate for the volume deficit before induction of general anesthesia^11,28^. In group L, the patients consistently received an infusion of balanced crystalloids at a rate of 10 ml/kg/h. Following this, the induction of general anesthesia was initiated 30 min later. This protocol for patients of group L aimed to offset the physiological fluid loss induced by preoperative fasting and fluid restriction appropriately^29^. Carotid FTc values, SBP, DBP, MAP, and HR were recorded before and after rehydration. SBP, DBP, MAP, and HR were recorded at 1, 2, and 3 min after induction, and the lowest value was taken. We further divided the 112 patients included in the Part I study into group L1 (carotid FTc ≥334.95 ms, *n*=47) and group M1 (carotid FTc <334.95 ms, *n*=65) according to pre-induction carotid FTc values and compared the incidence of post-induction hypotension between four groups (Fig. 1).

### Methods for ultrasound measurement of carotid FTc

**Figure 2:**
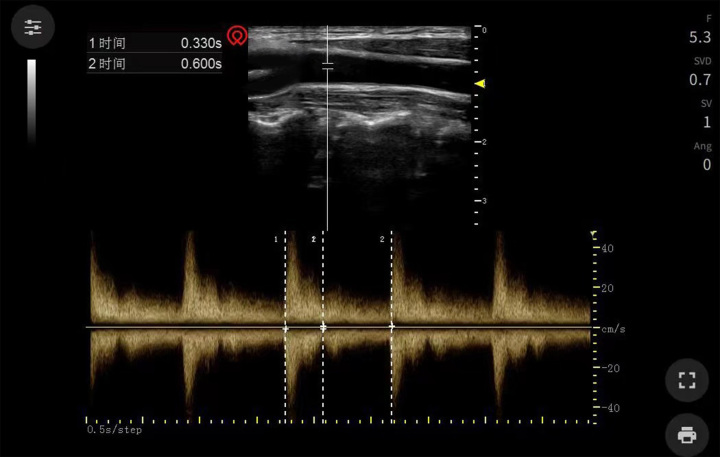
Carotid artery pulsed Doppler flow spectrograms.

Carotid FTc was measured using carotid ultrasonography as previously described^23^. The patient’s head was positioned in a supine position without a pillow and rotated 30° to the left. The color Doppler ultrasound instrument was used with a frequency conversion linear array probe with a frequency range of 6.0–13.0 MHz. The probe was positioned longitudinally on the patient’s neck, with the probe mark facing toward the patient’s head. A real-time B-ultrasound image was obtained of the long axis of the right common carotid artery from the lower edge of the thyroid cartilage. Subsequently, the sampling line was positioned at the center of the carotid lumen, ~2 cm away from the carotid bifurcation, in order to obtain the carotid pulse Doppler flow spectrum (Fig. 2) and record the carotid blood flow waveform. The caliper function of the ultrasound instrument was used to measure the interval between the ascending systolic phase and the dicrotic notch in order to obtain the carotid artery flow time (FT). In this study, the Wodey’s (W) formula^30^ was utilized to calculate the FTc, expressed as FTc=FT + [1.29 × (HR−60)].

### Statistical analysis

Statistical analysis was conducted using SPSS 26.0 software. Measures were reported as mean ± standard deviation for normally distributed data, median (interquartile range) for non-normally distributed data, and count (percentage) for categorical data. Independent samples *t*-tests were used to test normally distributed measures, two-factor repeated measures analysis of variance (ANOVA) was used for comparisons of continuous variables at different time points, and *χ*² test or Fisher’s exact test was used for count data. ROC analysis was performed to assess the reliability of carotid FTc in predicting post-induction hypotension and determine the optimal cut-off value. Logistic regression analysis was conducted to identify risk factors associated with the occurrence or non-occurrence of hypotension following general anesthesia induction in elderly patients. Two-tailed tests were used for hypothesis testing, and *P*<0.05 was considered statistically significant.

## Results

### Part I: To investigate the predictive value of carotid FTc for hypotension after general anesthesia induction in elderly patients undergoing elective surgery

Part I of this study included 112 elderly patients, sorted into the hypotension group (HA, *n*=73) or the non-hypotension group (NHA, *n*=39), determined by the presence or absence of hypotension following induction. There were no differences in patient demographic and clinical baseline values, including age, BMI, sex, ASA status, history of hypertension, and baseline SBP, DBP, MAP, and HR (Table 1, *P*>0.05).

**Table 1:** Demographic and clinical baseline values of the participants enrolled in Part I study.

Parameter	All (*n*=112)	HA (*n*=73)	NHA (*n*=39)	*P*
Age (years)	69.82±4.26	70.07±4.37	69.36±4.07	0.404
BMI (kg/m^2^)	22.72±2.98	2 2.64±2.93	22.87±3.11	0.702
Sex (male/female)	68/44	43/30	25/14	0.592
ASA status (II/III)	96/16	62/11	34/5	0.746
Hypertension (with/without)	40/72	28/45	12/27	0.425
Baseline SBP (mmHg)	143.37±16.22	142.64±16.98	144.72±14.81	0.521
Baseline DBP (mmHg)	69.98±8.39	69.96±9.03	70.03±7.15	0.968
Baseline MAP (mmHg)	94.28±9.55	94.21±10.06	94.41±8.64	0.915
Baseline HR (beats/min)	73.69±10.88	74.89±11.75	71.44±8.73	0.110

DBP, diastolic blood pressure; HA, hypotension group; HR, heart rate; MAP, mean arterial pressure; NHA, non-hypotension group; SBP, systolic blood pressure.

**Figure 3:**
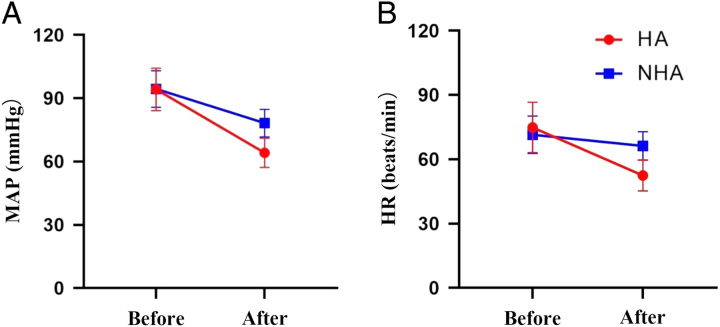
MAP and HR before and after induction in two groups of Part I study. HA, hypotension group; HR, heart rate; MAP, mean arterial pressure; NHA, non-hypotension group.

Hemodynamic variables before and after induction are shown in Table 2 and Fig. 3. The results of the repeated measures ANOVA demonstrated significant differences in MAP and HR between patients in the HA group and the NHA group (*P*<0.001). Additionally, significant differences were observed at each time point (*P*<0.001), and a cross-over effect was identified between the groups and time (*P*<0.001). These findings indicated that the overall level of MAP and HR in the HA group was lower than that in the NHA group. Following induction, both groups experienced a significant decrease in MAP and HR, but the decrease was more pronounced in the HA group.

**Table 2:** Mean arterial pressure and heart rate before and after induction in two groups of Part I study.

Parameter	Group	Before induction	After induction
MAP (mmHg)	HA (*n*=73)	94.21±10.06	64.16±6.90
	NHA (*n*=39)	94.41±8.64	78.21±6.55
HR (beats/min)	HA (*n*=73)	74.89±11.75	52.44±7.23
	NHA (*n*=39)	71.44±8.73	66.26±6.63

HA, hypotension group; HR, heart rate; MAP, mean arterial pressure; NHA, non-hypotension group.

**Figure 4:**
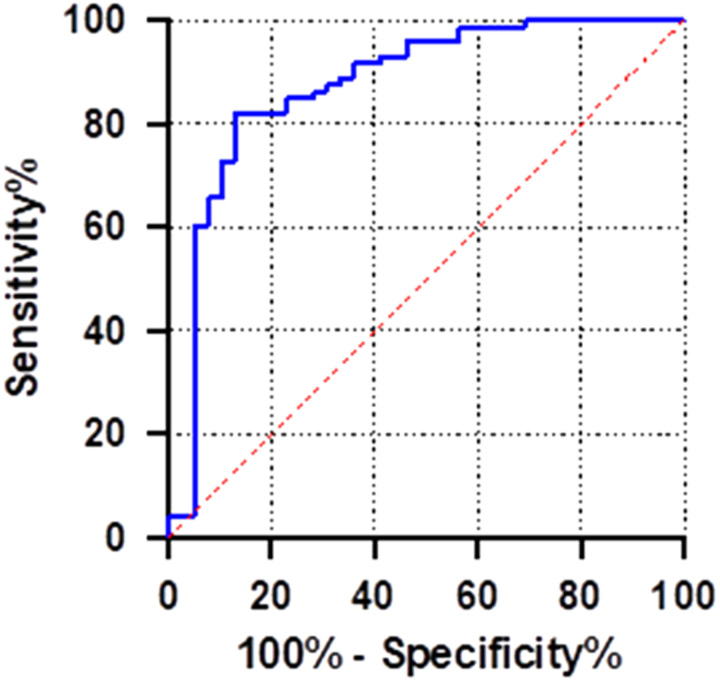
ROC curve of carotid FTc predicting the occurrence of post-induction hypotension in elderly patients with general anesthesia. FTc, corrected flow time; ROC, receiver operating characteristic.

Carotid FTc values prior to the induction of general anesthesia were significantly lower in the HA group compared to the NHA group (Table 3, *P*<0.001). The analysis of the ROC results, as presented in Table 4 and Figure 4, indicated that carotid FTc has a high predictive value for hypotension following the induction of general anesthesia in elderly patients. The AUROC was determined to be 0.876 [95% confidence interval (CI): 0.800–0.952, *P*<0.001]. The optimal cut-off value was identified as 334.95 ms, with a sensitivity of 87.20% and specificity of 82.20%.

**Table 3:** Comparison of the carotid corrected flow time measurements before induction in the two groups of Part I study.

Parameter	HA (*n*=73)	NHA (*n*=39)	*P*
Carotid FTc (ms)	323.21±15.11	346.20±21.11	<0.001

FTc, corrected flow time; HA, hypotension group; NHA, non-hypotension group.

**Table 4:** Diagnostic performance of carotid corrected flow time to predict post-induction hypotension.

	AUROC curve (95% CI)	*P*-value	Optimal cut-off value^*^	Sensitivity (%)	Specificity (%)	Youden index
FTc	0.876 (0.800–0.952）	<0.001	334.95 ms	87.20	82.20	0.694

* Optimal cut-off value was determined by maximising the Youden index.

AUROC, area under the receiver operating characteristic curve; CI, confidence interval; FTc, corrected flow time.

Logistic regression analysis was conducted to identify the risk factors associated with the development of hypotension following the induction of general anesthesia in elderly patients. Table 5 presented the results of logistic univariate analysis, which examined the variables of sex, age, ASA status, BMI, history of hypertension, carotid FTc, and baseline SBP, DBP, MAP, and HR. The analysis revealed that carotid FTc was a significant risk factor for hypotension after the induction of general anesthesia in elderly patients, with an odds ratio (OR) value of 0.895. Moreover, the results of logistic multivariate analysis demonstrated that carotid FTc independently served as a prognostic factor for hypotension induced by general anesthesia in the elderly, acting as a protective factor.

**Table 5:** Logistic regression analysis results.

	Univariate				Multivariate			
	Exp(B)	95% CI		*P*	Exp(B)	95% CI		*P*
Sex	0.803	0.359	1.792	0.592	–	–	–	–
Age (years)	1.042	0.947	1.147	0.401	–	–	–	–
ASA	1.206	0.387	3.760	0.746	–	–	–	–
BMI	0.975	0.855	1.111	0.699	–	–	–	–
Hypertension	1.400	0.612	3.203	0.426	–	–	–	–
Carotid FTc	0.895	0.853	0.937	<0.001	0.896	0.854	0.941	<0.001
Baseline SBP	0.992	0.968	1.016	0.518	–	–	–	–
Baseline DBP	0.999	0.954	1.047	0.968	–	–	–	–
Baseline MAP	0.998	0.958	1.039	0.914	–	–	–	–
Baseline HR	1.031	0.993	1.070	0.112	–	–	–	–

CI, confidence interval; DBP, diastolic blood pressure; FTc, corrected flow time; HR, heart rate; MAP, mean arterial pressure; SBP, systolic blood pressure.

### Part II: To explore the value of carotid FTc combined with perioperative fluid therapy in preventing hypotension after general anesthesia induction in elderly patients

Part II of this study incorporated 65 elderly patients who were categorized into two distinct groups based on their pre-induction carotid FTc values: group M (carotid FTc <334.95 ms, *n*=42) and group L (carotid FTc ≥334.95 ms, *n*=23). There were no differences in patient demographic and clinical baseline values, including age, BMI, sex, ASA status, history of hypertension, and baseline SBP, DBP, MAP, and HR (Table 6, *P*>0.05).

**Table 6:** Demographic and clinical baseline values of the participants enrolled in Part II study.

Parameter	All (*n*=65)	Group L (*n*=23)	Group M (*n*=42)	*P*
Age (years)	69.48±3.83	69.78±3.86	69.31±3.85	0.638
BMI (kg/m^2^)	22.28±2.47	22.17±2.72	22.33±2.34	0.805
Sex (male/female)	39/26	15/8	24/18	0.525
ASA status (II/III)	63/2	23/0	40/2	1.000
Hypertension (with/without)	34/31	15/8	19/23	0.123
Baseline SBP (mmHg)	142.32±14.38	139.91±12.07	143.64±15.48	0.321
Baseline DBP (mmHg)	73.63±8.58	75.17±10.24	72.79±7.71	0.293
Baseline MAP (mmHg)	96.54±9.07	96.83±9.17	96.38±9.13	0.852
Baseline HR (beats/min)	72.95±10.58	69.83±8.50	74.67±11.28	0.078

DBP, diastolic blood pressure; HR, heart rate; MAP, mean arterial pressure; SBP, systolic blood pressure.

**Figure 5:**
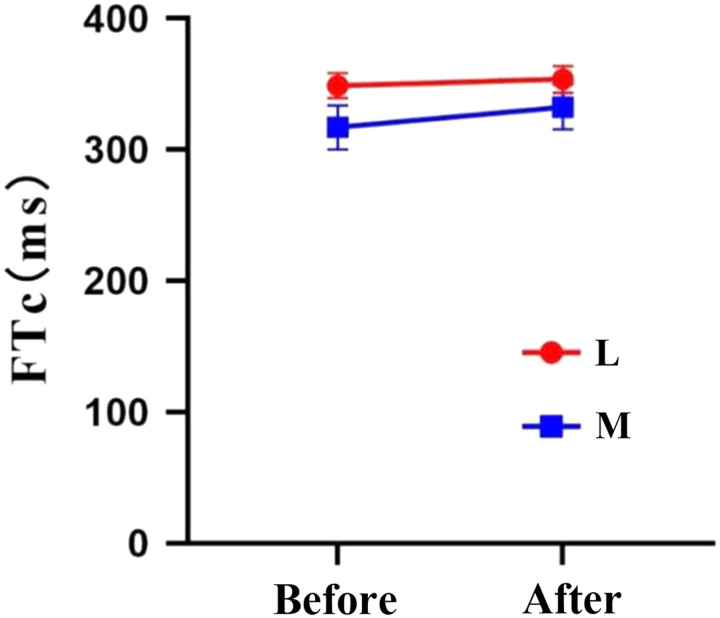
Comparison of carotid FTc before and after rehydration between two groups of patients. FTc, corrected flow time.

Patients in group M received significantly higher pre-induction fluid volume of rehydration compared to group L as a result of perioperative fluid therapy (Table 7, *P*<0.001), which was accompanied by a greater increase in carotid FTc levels among patients in group M following fluid therapy compared with patients in group L by repeated measures ANOVA (Table 8, Fig. 5).

**Table 7:** Comparison of the differences in the volume of fluid intake before induction of general anesthesia between patients in groups L and M.

Parameter	Group L (*n*=23)	Group M (*n*=42)	*P*
Fluid intake before induction (ml)	290.43±49.50	469.52±59.72	<0.001

**Table 8:** Carotid corrected flow time before and after rehydration between patients in groups L and M.

Parameter	Group	Before rehydration	After rehydration
Carotid FTc (ms)	Group L (*n*=23)	348.97±9.39	353.76±10.18
	Group M (*n*=42)	317.05±16.85	332.64±17.14

FTc, corrected flow time.

**Figure 6:**
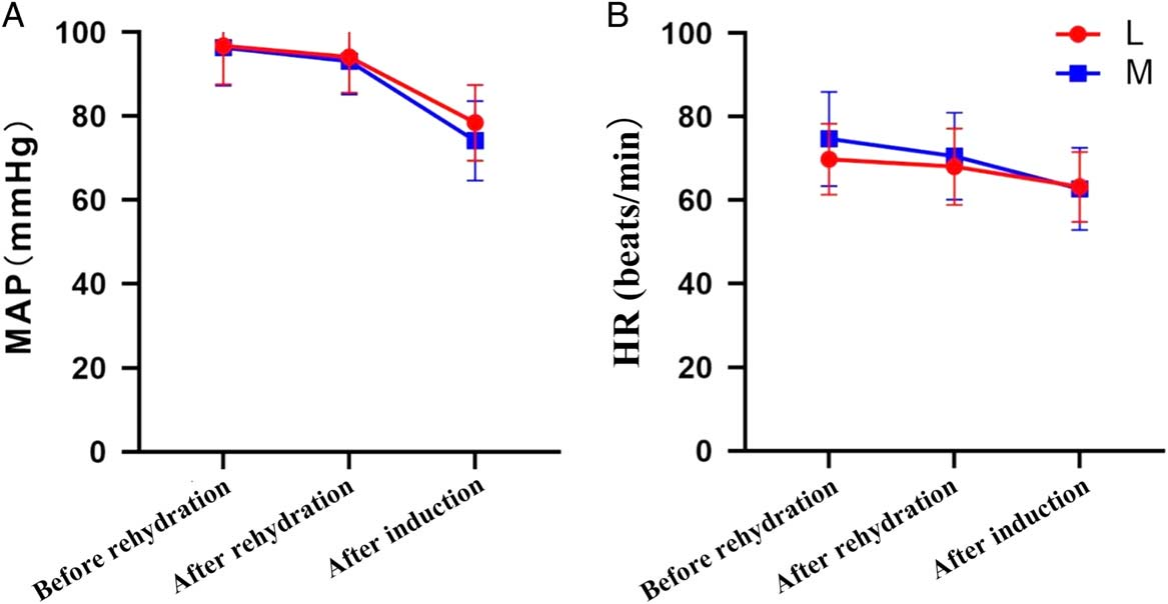
Changes of MAP and HR before and after rehydration, and after induction between patients in groups L and M. HR, heart rate; MAP, mean arterial pressure

Hemodynamic variables before and after rehydration and after induction are shown in Table 9 and Figure 6. The results of the repeated measures ANOVA indicated no significant differences in MAP and HR between patients in group M and group L (*P*>0.05). Furthermore, no statistically significant differences were observed at each time point (*P*>0.05), and there was no evidence of a cross-over effect between the groups and time (*P*>0.05). These findings suggested that the levels of MAP and HR were comparable between the two groups at all time points, and they were similarly reduced following the induction of anesthesia. Moreover, we found no statistically significant difference in the incidence of hypotension after induction of general anesthesia between patients in groups L and M (Table 10, *P*>0.05).

**Table 9:** Changes of mean arterial pressure and heart rate between patients in groups L and M.

Parameter	Group	Before rehydration	After rehydration	After induction
MAP (mmHg)	Group L (*n*=23)	96.83±9.17	94.09±8.45	78.43±9.04
	Group M (*n*=42)	96.38±9.13	93.10±7.93	74.10±9.51
HR (beats/min)	Group L (*n*=23)	69.83±8.50	68.00±9.15	63.17±8.34
	Group M (*n*=42)	74.67±11.28	70.52±10.42	62.69±9.81

HR, heart rate; MAP, mean arterial pressure.

**Table 10:** Comparison of the incidence of hypotension after induction of general anesthesia in patients of groups L and M.

Parameter	Group L (*n*=23)	Group M (*n*=42)	*P*
Hypotension after induction (%)	30.43	35.71	0.667

In order to further investigate the impact of perioperative fluid therapy on preventing hypotension following the induction of general anesthesia in elderly patients, we divided the 112 patients included in Part I study into group L_1_ (carotid FTc ≥334.95 ms, *n*=47) and group M_1_ (carotid FTc <334.95 ms, *n*=65) according to pre-induction carotid FTc values. There were no differences in patient demographic and clinical baseline values in four groups, including groups L_1_, M_1_, L, and M (Table 11, *P*>0.05). Importantly, our observations revealed that, despite the patients in group L receiving a continuous infusion of balanced crystalloids at a rate of 10 ml/kg/h for a span of 30 min, designed to offset the physiological fluid loss induced by preoperative fasting and fluid restriction appropriately, there was no significant variance in the incidence of post-induction hypotension when compared to patients in group L_1_ (Table 12, *P*>0.05). Conversely, in patients from the group M who received perioperative fluid therapy, the incidence of post-induction hypotension was notably lower compared to the group M_1_ (Table 13, *P*<0.001).

**Table 11:** Demographic and clinical baseline values of the participants in groups L_1_, M_1_, L, and M.

Parameter	Group L_1_ (*n*=47)	Group M_1_ (*n*=65)	Group L (*n*=23)	Group M (*n*=42)	*P*
Age (years)	69.57±3.97	70.00±4.49	69.78±3.86	69.31±3.85	0.856
BMI (kg/m^2^)	22.55±3.07	22.85±2.93	22.17±2.72	22.33±2.34	0.712
Sex (male/female)	29/18	39/26	15/8	24/18	0.930
ASA status (II/III)	42/5	54/11	23/0	40/2	0.066
Baseline MAP (mmHg)	94.83±9.62	93.88±9.56	96.83±9.17	96.38±9.13	0.446
Baseline HR (beats/min)	72.19±9.04	74.77±11.98	69.83±8.50	74.67±11.28	0.189

HR, heart rate; MAP, mean arterial pressure.

**Table 12:** Comparison of the incidence of hypotension after induction of general anesthesia in patients of groups L_1_ and L.

Parameter	Group L_1_ (*n*=47)	Group L (*n*=23)	*P*
Hypotension after induction (%)	27.66%	30.43%	1.000

**Table 13:** Comparison of the incidence of hypotension after induction of general anesthesia in patients of groups M_1_ and M.

Parameter	Group M_1_ (*n*=65)	Group M (*n*=42)	*P*
Hypotension after induction (%)	92.31%	35.71%	<0.001

## Discussion

Hypotension resulting from blood pressure fluctuation following the induction of general anesthesia is a prevalent adverse hemodynamic phenomenon, particularly in elderly patients^8^. Hypotension following the induction of general anesthesia in elderly patients can lead to organ system damage, including acute cerebral ischemia, acute myocardial infarction, acute kidney injury, and other severe complications^7,27,31^. It is also closely associated with delayed awakening after general anesthesia and an increase in perioperative mortality^32,33^. The prevention of post-induction hypotension in elderly patients is essential for promoting their prompt postoperative recovery and long-term regression. Therefore, accurately predicting and assessing the occurrence of post-induction hypotension in elderly patients undergoing general anesthesia and implementing appropriate therapeutic measures are of significant clinical importance. The results of this study demonstrated that pre-induction ultrasound measurement of carotid FTc is a reliable predictor for hypotension occurrence after induction of general anesthesia in elderly patients, with an AUROC of 0.876 and an optimal threshold of 334.95 ms. The carotid FTc less than 334.95 ms before anesthesia induction indicated a significant increase in the risk of post-induction hypotension. Importantly, administering perioperative fluid therapy with a fluid volume of 8 ml/kg performed 30 min prior to anesthesia induction for elderly patients with carotid FTc less than 334.95 ms before induction effectively reduced the incidence of post-induction hypotension. Thus, we firstly proposed that a combination of carotid FTc and perioperative fluid therapy at a specific fluid volume should be considered as an effective approach to reduce the incidence of hypotension during the perioperative period in elderly patients.

The primary factors contributing to hypotension following the induction of general anesthesia are the patient’s hypovolemic condition, cardiovascular depression, and the vasodilatory effects of anesthetics^34,35^. HR, in harmony with blood pressure, serves as a fundamental indicator reflecting patients’ hemodynamic conditions. It is also influenced by hypovolemia and anesthetic cardiovascular depression, particularly by sufentanil-induced central vagus nerve stimulation^35,36^. Throughout our study, a majority of patients exhibited a decline in HR and blood pressure following the induction of general anesthesia. Interestingly, in Part I of the trial, HR and MAP decreased more significantly in the HA group compared to the NHA group. These findings suggest that HR and blood pressure conjointly mirror the patient’s circulatory state. It is also evident that the degree of reduction in HR and blood pressure corresponds to the patient’s blood volume profile prior to induction. Special attention should be given to evaluating the preoperative volume status of patients, especially when other influencing factors cannot be modified. Previous researches^37,38^ have established that advanced age is an independent risk factor for hypotension following the initiation of general anesthesia. Elderly individuals have an inherent susceptibility to cardiovascular instability and hypotension due to factors such as a higher prevalence of left ventricular diastolic dysfunction, reduced vascular responsiveness, increased sensitivity to anesthetics, and longer periods of preoperative fasting and preparation, which may result in preoperative volume depletion and subsequent hypotension post-anesthesia. Furthermore, elderly patients exhibit decreased tolerance to post-induction hypotension and are prone to experiencing severe postoperative complications^39^. Consequently, this study holds significant clinical value in the exploration of approaches for predicting the occurrence of hypotension after general anesthesia in elderly patients.

In recent years, bedside ultrasound techniques have gained widespread clinical utilization in assessing patient volume status, predicting fluid responsiveness, and anticipating the occurrence of hypotension following the induction of general anesthesia^40,41^. However, venous ultrasound indices, such as the diameter of the inferior vena cava (IVC) and the IVC collapse index, may be less reliable in predicting fluid responsiveness in spontaneously breathing patients. This is due to their susceptibility to variations caused by patient breathing patterns^42^. In recent years, common carotid artery blood flow has become a prominent alternative method due to its non-invasive nature and reproducibility. Evidence from studies^40,41^ indicates that assessing the volume status of patients and predicting volume responsiveness during voluntary respiratory states can be accomplished using corrected carotid flow time obtained through common carotid artery flow spectroscopy. A recent study^23^ has shown the potential of ultrasound measurement of carotid FTc as a promising technique for evaluating preoperative volume status in patients, which remains unaffected by respiration and other factors and becomes a reliable predictor of volume responsiveness in spontaneously breathing patients. Thus, in this study, ultrasound measurement of carotid FTc was selected as a predictor of hypotension following the induction of general anesthesia in elderly patients. Maitra *et al*.^10^ reported that carotid FTc was found to predict post-induction hypotension in adult patients (aged 18–65 years) undergoing elective surgery with an ASA classification of I to II. The study showed an AUROC of 0.910, with an optimal cut-off value of 330.20 ms. Our study also demonstrated that carotid FTc has a high predictive value for hypotension following the induction of general anesthesia in elderly patients, with an AUROC of 0.876 and the optimal cut-off value of 334.95 ms. However, in the current study, the determined optimal cut-off value of 334.95 ms was found to be longer than the previously reported 330.20 ms. This difference may be attributed to the relatively longer left ventricular systolic period caused by slower HR in elderly individuals^43^.

Perioperative fluid therapy is an effective approach in preventing hypotension after the induction of general anesthesia^44,45^. This treatment protocol primarily utilizes two major categories of fluid solutions: crystalloids and colloids. The chosen fluid solution type may potentially influence patient outcomes. Among crystalloids, balanced solutions containing electrolytes and an acid–base balance close to that of plasma are increasingly preferred to 0.9% sodium chloride solution, which is wrongly named normal saline. Employing chlorine-rich solutions may precipitate hyperchloremic acidosis, renal vasoconstriction, and acute kidney injury. Conversely, utilizing balanced salt solutions like lactated Ringer’s solution reduces such risks^46^. Comprising larger molecules than crystalloids, colloids are anticipated to remain within the circulation longer. Colloids encompass dextrans, hydroxyethyl starches (HES), gelatins, and albumin^46,47^. While colloids may provide longer-lasting effective blood volume maintenance, they pose risks of coagulation dysfunction, renal damage, and allergic reactions in elderly patients^48–50^. Therefore, balanced crystalloids were chosen for perioperative fluid therapy in this study. In Part II of our study, we observed no discernible disparity in the incidence of hypotension between groups L and M. Further scrutiny of the patient data from both Parts I and II revealed a lack of statistically significant divergence in the rate of hypotension post-induction between groups L and L_1_. Conversely, the rate of post-induction hypotension in group M was significantly lower than in group M_1_. These findings insinuated that additional factors^34,35^ might contribute to hypotension following the induction of general anesthesia. Explicitly, suitable fluid infusion conducted pre-induction in patients demonstrating sufficient volume does not impact the occurrence of post-induction hypotension. Conversely, in patients exhibiting insufficient pre-induction volume, perioperative fluid therapy involving a certain volume of fluid can noticeably mitigate occurrence of post-induction hypotension. It was also observed that group M, which underwent perioperative fluid therapy before induction, experienced a greater increase in FTc values before and after rehydration compared to group L. This difference was attributed to the amount of rehydration received. However, elderly patients with multiple comorbidities exhibit limited tolerance to rapid fluid administration due to degenerative changes and organ lesions in the heart, kidneys, and other organs^51^. Therefore, there is a need to further investigate how to precisely control the amount and speed of perioperative fluid therapy before induction of general anesthesia to avoid volume overload and exacerbation of cardiac burden.

In this study, we acknowledge several limitations. Firstly, elderly patients inherently possess a heightened risk for atherosclerosis and peripheral vascular disease (PVD). Despite rigorous exclusion of patients with these conditions through careful medical history review and initial physical assessments performed by a seasoned vascular surgeon, the residual possibility of undetected PVD and atherosclerosis in the enrolled patients persists. Such condition potentially influences the accuracy of carotid FTc in predicting fluid responsiveness. Accordingly, future studies should consider comprehensive examinations for PVD and atherosclerosis. Secondly, this study did not focus on a specific type of surgery, and patients subject to various surgical procedures carry distinct disease states, which may potentially skew the study findings and compromise the accuracy of the outcomes. To ensure greater reliability, future studies will exclusively investigate elderly patients undergoing a specific type of surgery. Various prognostic indicators will be monitored post-surgery to investigate the potential role of carotid FTc combined with perioperative fluid therapy in patients with certain disorders. Specifically, we aim to discern whether reducing the incidence of hypotension post-induction could enhance patient prognosis. Additionally, in this study, crystalloid fluid was utilized for perioperative fluid therapy. However, it is noteworthy that colloidal fluid may have a longer-lasting effect on maintaining effective blood volume. Nevertheless, its usage in elderly patients may be associated with potential risks such as coagulation disorders, renal impairment, and allergic reactions. Assessing the safety and efficacy of crystalloid and colloid fluids will be a key focus in future studies. Furthermore, the relatively short time period from patient admission to the operating theater until the induction of general anesthesia raises concerns about administering rapid and large volumes of balanced crystalloids for perioperative fluid therapy, particularly in elderly patients who may be vulnerable to complications such as acute heart failure. Although none of the patients in our study displayed signs of volume overload following perioperative fluid therapy, we did not conduct postoperative follow-up on their cardiopulmonary function. Longer and slower perioperative fluid therapy has been found to be more advantageous for elderly patients, particularly those at high risk with underlying cardiovascular conditions. Consequently, our future investigations will focus on exploring different lengths of time and speed gradients for perioperative fluid therapy, aiming to establish optimal approaches suitable for specific elderly populations before the induction of general anesthesia. Through this endeavor, we aim to enhance perioperative management strategies and facilitate accelerated postoperative recovery for patients.

## Conclusion

In conclusion, our study demonstrated that carotid FTc serves as a reliable predictor for hypotension resulting from blood pressure fluctuation after induction of general anesthesia in elderly patients, and the implementation of perioperative fluid therapy is instrumental in significantly reducing the incidence of hypotension after the induction of general anesthesia in elderly patients with carotid FTc values below 334.95 ms prior to anesthesia induction. These findings demonstrate that the combination of carotid FTc and perioperative fluid therapy holds promise as an important method in clinical anesthesia for preventing the occurrence of hypotension after the induction of general anesthesia in elderly patients.

## Ethical approval

This study received approval from the Medical Ethics Committee of Fujian Medical University Union Hospital (approval number: 2022YF046-01) and was registered in the ClinicalTrials Research Registry (registration number: 2022XHYG0018-01). (https://classic.clinicaltrials.gov/ct2/show/NCT05628051?term=2022XHYG0018-01&draw=2&rank=1).

## Consent

All participants provided informed consent, either personally or through their family members, by signing an informed consent form.

## Sources of funding

The study was supported by grants from the Key Clinical Specialty Discipline Construction Program of Fujian Medical University Union Hospital (0252004).

## Author contribution

L.Z.: concept and design; S.H., Z.L., A.C., J.W., and X.X.: acquisition, analysis, or interpretation of data; S.H., Z.L., and A.C.: drafting of the manuscript; L.Z.: critical revision of the manuscript for important intellectual content; S.H., Z.L., and A.C.: statistical analysis.

## Conflicts of interest disclosure

The authors declare no conflicts of interest.

## Research registration unique identifying number (UIN)

1. Name of the registry: ClinicalTrials Research Registry.
2. Unique identifying number or registration ID: 2022XHYG0018-01.
3. Hyperlink to your specific registration (must be publicly accessible and will be checked): https://classic.clinicaltrials.gov/ct2/show/NCT05628051?term=2022XHYG0018-01&draw=2&rank=1.

## Guarantor

Liangcheng Zhang, Shishi Huang, Zhenqi Liao, and Andi Chen.

## Data availability statement

The datasets utilized and/or analyzed in this study can be obtained from the corresponding author upon a reasonable request.

## Provenance and peer review

Not commissioned, externally peer-reviewed.
